# A Nonlinear Maximum Correntropy Information Filter for High-Dimensional Neural Decoding

**DOI:** 10.3390/e23060743

**Published:** 2021-06-12

**Authors:** Xi Liu, Shuhang Chen, Xiang Shen, Xiang Zhang, Yiwen Wang

**Affiliations:** 1 Department of Electronic and Computer Engineering, Hong Kong University of Science and Technology, Hong Kong, China; lxi1102@163.com (X.L.); xshenai@connect.ust.hk (X.S.); xzhangaz@ust.hk (X.Z.); 2 Department of Chemical and Biological Engineering, Hong Kong University of Science and Technology, Hong Kong, China; schenbx@connect.ust.hk

**Keywords:** state-observation model, high-dimensional measurements systems, correntropy

## Abstract

Neural signal decoding is a critical technology in brain machine interface (BMI) to interpret movement intention from multi-neural activity collected from paralyzed patients. As a commonly-used decoding algorithm, the Kalman filter is often applied to derive the movement states from high-dimensional neural firing observation. However, its performance is limited and less effective for noisy nonlinear neural systems with high-dimensional measurements. In this paper, we propose a nonlinear maximum correntropy information filter, aiming at better state estimation in the filtering process for a noisy high-dimensional measurement system. We reconstruct the measurement model between the high-dimensional measurements and low-dimensional states using the neural network, and derive the state estimation using the correntropy criterion to cope with the non-Gaussian noise and eliminate large initial uncertainty. Moreover, analyses of convergence and robustness are given. The effectiveness of the proposed algorithm is evaluated by applying it on multiple segments of neural spiking data from two rats to interpret the movement states when the subjects perform a two-lever discrimination task. Our results demonstrate better and more robust state estimation performance when compared with other filters.

## 1. Introduction

Brain machine interface (BMI) establishes a direct communication pathway between the brain and external devices [1,2,3,4,5,6]. BMI collects the noisy signals from hundreds of neurons in the brain, and estimates a motor intention from these signals [7,8]. This estimated movement intention can then be used to control the robot to assist motor disabled people [9,10,11,12,13,14,15,16,17,18,19]. Signal processing algorithms play a key role in BMI. As a commonly-used state-observation model, the Kalman filter (KF) has been adopted to decode the movement intents as the state from the high-dimensional observations formed by multiple neural firing activity [20,21,22,23], in which the movement state evolves over time as described by the linear state model and the observation model reflects how the neuron firing tunes to movement with Gaussian noise. The implementation of the Kalman filter nicely considers the gradual change of the continuous brain state, and thus is especially appropriate for the brain control task where the subject continuously adjusts the brain states to control an external robot.

However, there are some challenges in applying the state-observation model for BMI. Firstly, the nervous systems are nonlinear in general [24,25], and the traditional KF is not fit for nonlinear systems. As the extensions of KF, the extended Kalman filter (EKF) [26], unscented Kalman filter (UKF) [27,28], and cubature Kalman filter (CKF) [29] can deal with a nonlinear system. However, these algorithms can only approximate the nonlinear function by a finite-order level of its Taylor expansion and lead to an inaccurate state estimation. Secondly, the motor state is controlled by hundreds of neurons, whose activity could be highly connected [30,31,32]. The components of measurements in traditional KF decoding are assumed to be conditionally independent to update the states, and some connections among these components, e.g., the neural connectivity, may exist in the experiments of BMI that cannot be reflected in the traditional KF. Discriminative Kalman filter (DKF) was applied to decode movement states from high-dimensional neural firings [19]. DKF computes the posterior of the state-given observation by approximating only the mean and variance, which may not be accurate for an arbitrary nonlinear system. The key goal in BMI is to accurately estimate the motor state in real-time through measuring the noisy activities of many neurons that tune nonlinearly and correlatedly to the states. Thirdly, the traditional KF may not be an ideal tool for real-time robust decoding to address the high-dimensional measurement data. This is because the Kalman gain computes the inverse of a correlation matrix with the same dimension of measurements, and the high dimensionality brings a heavy computational burden for online applications. In addition, KF relies strongly on good initial conditions and is not robust to the outliers in the observation. It brings problems to robust online decoding. In particular, the initial movement states in BMI cannot always be known, for example, the limb status of paralyzed patients may not exist. It could be difficult to set the proper initial conditions as well as the uncertainty reflected in the covariance matrix. For online decoding, the performance of the Kalman filter could be significantly affected by outliers in the observation, such as recording noise in neural activity.

We are interested in addressing the nonlinearity and existing connectivity among high-dimensional observations for robust neural decoding. The neural network (NN) has demonstrated an arbitrary nonlinear regression ability in modeling [33], and has been used to decode the motor intention from multiple neuron activities [34,35,36]. The advantage of using NN is to allow arbitrary dependence among the neurons and provide a good method to reduce the dimensionality from high-dimensional neurons to low-dimensional states. However, a simple multi-layer perception (MLP) network could ignore the time dynamics in brain states. The recurrent neural network (RNN) model considers the possible internal dynamics and has been used to decode the letter shapes for classification [37]. The long short-term memory (LSTM) network has been used in the classification of the motor imagery task based on electroencephalography (EEG) signals [38]. For the brain control in motor BMI, where we adopt invasive signals to decode continuous motor intention, the Kalman filter has been a more popular decoder [21,23,39,40]. The information filter [41] was proposed to address the sensitivity to the initial conditions and reduce the computational complexity, in which the inverse of the covariance matrix defining the uncertainty is propagated instead. However, the current information filter is also only designed for simple linear systems. To address the robustness of the filtering with respect to the outliers, i.e., heavy-tailed non-Gaussian noises [42], the traditional minimum mean square error (MMSE) criterion should be reconsidered. As an alternative, the correntropy theory in information theoretic learning (ITL) is an effective tool, which captures all even-order statistical information rather than the traditional second-order statistics [43,44,45]. In particular, its optimization criterion, called maximum correntropy criterion (MCC), has been extensively used in robust filters [46,47,48,49,50,51,52], mostly for linear systems.

In this paper, we propose a nonlinear maximum correntropy information filter (NMCIF) to robustly estimate the state from noisy high-dimensional measurements for the nonlinear systems. We utilize the universal nonlinear approximator neural network to preprocess the high-dimensional measurements, which allows dependence among the measurements to be considered. We introduce correntropy as the optimization criterion to improve the robustness of the filter with respect to heavy-tailed non-Gaussian noises. The uncertainty of the state estimation is derived and propagated over time to reduce the computational burden and the sensitivity to the initial conditions. The preferred information matrix and the proof of the robustness of the algorithm are given, and the convergence speed is analyzed theoretically. The proposed algorithm is applied to real neural recording from two rats while the subjects were manually controlling a two-lever discrimination task. The two-dimensional movement states are estimated from multiple neuronal activities. The performance, convergence time, the sensitivity to the bad initial conditions, and robustness to heavy-tailed noise of the proposed algorithm are compared with those of the traditional Kalman filter and neural network decoder. We also use the mean square error in 2D to evaluate the movement estimation compared with the ground truth.

Our contribution is to propose a new methodology to better estimate the state in the filtering process for the noisy high-dimensional measurement systems. The neural network is utilized to construct the nonlinear measurement model between the high-dimensional measurements and low-dimensional states. The correntropy criterion is considered in deriving the state to cope with the non-Gaussian noise and eliminate uncertainty. The rest of this paper is organized as follows. In Section 2, the NMCIF is introduced. In Section 3, the detailed derivation of NMCIF is given, and the robustness and convergence of the algorithm are analyzed. In Section 4, the real data application on the two-lever discrimination task of rats is used to validate the effectiveness of the proposed algorithm compared with other filters. In Section 5, the discussion and conclusion are given.

## 2. Nonlinear Maximum Correntropy Information Filter

In general, the state-observation model is written as dynamic systems:

(1) $$x_{k}=F\left(x_{k-1}\right)+q_{k-1},$$

(2) $$y_{k}=H\left(x_{k}\right)+\zeta_{k},$$

where $x_{k}$ denotes the *n*-dimensional state at time *k*. In our application, it is the 2-dimensional position. *F* denotes the state transition function. In our application, it is a linear function F estimated from the training data by least square [53]. $q_{k}$ denotes the process noise with mean ${\bar{q}}_{k}$ and covariance matrix $Q_{k}$, which is estimated by the residue of the state transition approximation. $y_{k}$ denotes the *m*-dimensional measurements. In our application, it is a high-dimensional vector formed by multiple neural firing activities considering the firing history. *H* is the nonlinear observation model. In our application, it is estimated from the state vector to each dimension of the neural activity independently. In the linear case, each row of *H* describes how the neural activity weighted tunes the movement states. $\zeta_{k}$ denotes the measurement noise, which is estimated by the residue of using the observation model. For a nonlinear system with high-dimensional correlated observation, such as the most experiments of BMI, the noisy measurements would be collected from hundreds of neurons, and there exists heavy connectivity among neurons.

The commonly-used second-order statistics method, the MMSE criterion, may not be optimal as the nonlinear system generally does not have Gaussian distributed noise [42]. Correntropy is a key concept in ITL, which measures the similarity between two random variables considering all the even order statistics. Assuming *X* and *Y* are two random variables, the correntropy is defined as:

(3) $$V\left(X,Y\right)=E\left[\kappa (X,Y)\right]=\int \kappa \left(x,y\right)dF_{XY}\left(x,y\right),$$

where E represents the expectation operator, $F_{XY}\left(x,y\right)$ denotes the joint distribution function with respect to the two random variables, and $\kappa \left(\cdot ,\cdot \right)$ is a shift-invariant Mercer kernel. Gaussian kernel is simple and has been successfully applied in a heavy-tailed noise environment [44,46,47], and we chose the Gaussian kernel to eliminate this noise, i.e.,

(4) $$\kappa \left(x,y\right)=G_{\sigma }\left(e\right)=\exp\left(-\frac{e^{2}}{2\sigma^{2}}\right),$$

where e=x−y, and σ>0 is the kernel bandwidth. Taking the Taylor series expansion of the Gaussian kernel, it is noted that the correntropy contains not only the second-order moment but also all higher even order statistical information. In particular, when the kernel bandwidth σ is 20 times larger than the values chosen based on Silverman’s rule [44], the second-order moment plays a key role in correntropy.

In this section, we propose a new method for the state-observation model estimation. Let $W_{k}$ be defined as the information matrix of process noise, which is the inverse of the covariance matrix $W_{k}=Q_{k}^{-1}$. We propose the NMCIF.

The prior mean ${\bar{x}}_{k}$ and corresponding information matrix ${\bar{\chi }}_{k}$ are firstly computed as:

(5) $${\bar{x}}_{k}=F{\hat{x}}_{k-1},$$

(6) $${\bar{\chi }}_{k}=W_{k-1}-W_{k-1}F{\left({\hat{\chi }}_{k-1}+F^{T}W_{k-1}F\right)}^{-1}F^{T}W_{k-1}.$$

and the posterior mean ${\hat{x}}_{k}$ is recursively calculated by the fixed-point iterative methods [54] as:

(7) $${\hat{x}}_{k}^{\left(t\right)}={\bar{x}}_{k}+{\tilde{K}}_{k}^{\left(t-1\right)}\left(g\left(y_{k}\right)-{\bar{x}}_{k}\right),$$

where $g\left(\cdot \right)$ denotes the nonlinear preprocess on the observation that deducts the dimensionality of original observation into the new observation with the same dimension as the state, such that:

(8) $$g\left(y_{k}\right)=x_{k}+r_{k}.$$

Superscript (t) stands for the fixed-point iteration index and the iterative process stops when:

(9) $$\frac{∥{\hat{x}}_{k}^{\left(t\right)}-{\hat{x}}_{k}^{\left(t-1\right)}∥}{∥{\hat{x}}_{k}^{\left(t-1\right)}∥}⩽\omega ,$$

with ω being a small positive value, or the iterative index reaches a preset value. ${\tilde{K}}_{k}^{\left(t-1\right)}$ stands for the Kalman gain as:

(10) $${\tilde{K}}_{k}^{\left(t-1\right)}={\left({\tilde{\chi }}_{k}^{\left(t-1\right)}+{\tilde{V}}_{k}^{\left(t-1\right)}\right)}^{-1}{\tilde{V}}_{k}^{\left(t-1\right)}.$$

Here ${\tilde{\chi }}_{k}^{\left(t-1\right)}$ is the revised prior information matrix denoted as in Equation (12) and ${\tilde{V}}_{k}^{\left(t-1\right)}$ is denoted as in Equation (13).

In our application, we use multi-layer perception as a universal approximator to take the high-dimensional neural firing as input and output for the preprocessed states as the observation. Then, the observation model is an identity matrix. $r_{k}$ denotes the neural network approximation residue with mean ${\bar{r}}_{k}$ and covariance matrix $R_{k}$. Since the neural network could consider the bias, we then assume the process noise and measurement noise have zero means. $V_{k}$ is defined by $V_{k}=R_{k}^{-1}$.

The corresponding information matrix ${\hat{\chi }}_{k}$ of the posterior is updated as:

(11) $${\hat{\chi }}_{k}={\tilde{\chi }}_{k}^{\left(t-1\right)}+{\tilde{V}}_{k}^{\left(t-1\right)},$$

where for each iteration *t*,

(12) $${\tilde{\chi }}_{k}^{\left(t-1\right)}=S_{\chi ,k}C_{\chi ,k}^{\left(t-1\right)}S_{\chi ,k}^{T},$$

(13) $${\tilde{V}}_{k}^{\left(t-1\right)}=S_{V,k}C_{V,k}^{\left(t-1\right)}S_{V,k}^{T},$$

(14) $$C_{\chi ,k}^{\left(t-1\right)}=\mathrm{diag}\left(G_{\sigma }\left(e_{k,1}^{\left(t-1\right)}\right),\ldots ,G_{\sigma }\left(e_{k,n}^{\left(t-1\right)}\right)\right),$$

(15) $$C_{V,k}^{\left(t-1\right)}=\mathrm{diag}\left(G_{\sigma }\left(e_{k,n+1}^{\left(t-1\right)}\right),\ldots ,G_{\sigma }\left(e_{k,2n}^{\left(t-1\right)}\right)\right),$$

(16) $$e_{k,i}^{\left(t-1\right)}=d_{k,i}-m_{k,i}{\hat{x}}_{k}^{\left(t-1\right)},$$

where $d_{k,i}$ denotes the *i*-th element of $D_{k}$, $m_{k,i}$ denotes the *i*-th row of $M_{k}$, and $D_{k}=S_{k}\left[\begin{array}{c} {\bar{x}}_{k}\\ g(y_{k}) \end{array}\right]$, $M_{k}=S_{k}\left[\begin{array}{c} I_{n}\\ I_{n} \end{array}\right]$, $e_{k}=S_{k}\delta_{k}$. Here we take $D_{k}$ as the transformed observation, $M_{k}$ is the new observation model, and $e_{k}$ is the noise term. $S_{k}$ can be obtained by making Cholesky decomposition on the inverse of covariance of the noise term $\delta_{k}=\left[\begin{array}{c} -\left(x_{k}-{\bar{x}}_{k}\right)\\ r_{k} \end{array}\right]$, and denoted as $S_{k}=\left[\begin{array}{cc} S_{\chi ,k} & 0\\ 0 & S_{V,k} \end{array}\right]$.

Note that when the kernel bandwidth becomes increasingly larger, the performance of the algorithm will behave like the corresponding nonlinear information filter (NIF). Especially, if σ→∞, then $C_{\chi ,k}\to I$ and $C_{V,k}\to I$, the proposed algorithm will reduce to the NIF. Thus, the NIF algorithm is a special case of the proposed algorithm, and is summarized as follows:

(17) $${\bar{x}}_{k}=F{\hat{x}}_{k-1},$$

(18) $${\bar{\chi }}_{k}=W_{k-1}-W_{k-1}F{\left({\hat{\chi }}_{k-1}+F^{T}W_{k-1}F\right)}^{-1}F^{T}W_{k-1},$$

(19) $$K_{k}={\left({\bar{\chi }}_{k}+V_{k}\right)}^{-1}V_{k},$$

(20) $${\hat{x}}_{k}={\bar{x}}_{k}+K_{k}\left(g\left(y_{k}\right)-{\bar{x}}_{k}\right),$$

(21) $${\hat{\chi }}_{k}={\bar{\chi }}_{k}+V_{k}.$$

## 3. Algorithm Derivation and Analysis

### 3.1. Derivation of Estimation on the Mean of Posterior

Next, we give a derivation process with respect to the nonlinear maximum correntropy information filter.

The mean of the prior state ${\bar{x}}_{k}$ and the corresponding information matrix are computed the same as Equations (5) and (6). By Equations (1), (8) and (17), we can build the following equation:

(22) $$\left[\begin{array}{c} {\bar{x}}_{k}\\ g\left(y_{k}\right) \end{array}\right]=\left[\begin{array}{c} I_{n}\\ I_{n} \end{array}\right]x_{k}+\delta_{k},$$

where $I_{n}$ is an n×n identity matrix, and $\delta_{k}=\left[\begin{array}{c} -(x_{k}-{\bar{x}}_{k})\\ r_{k} \end{array}\right]$.

We can easily obtain the inverse of covariance of the noise term in Equation (22) as the following equation:

(23) $$\begin{array}{cc} {\left(E\left[\delta_{k}\delta_{k}^{T}\right]\right)}^{-1} & =\left[\begin{array}{cc} {\bar{\chi }}_{k} & 0\\ 0 & V_{k} \end{array}\right]\\ \; & =\left[\begin{array}{cc} S_{\chi ,k}S_{\chi ,k}^{T} & 0\\ 0 & S_{V,k}S_{V,k}^{T} \end{array}\right]=S_{k}S_{k}^{T}, \end{array}$$

where $S_{k}$ is the Cholesky decomposition of ${\left(E\left[\delta_{k}\delta_{k}^{T}\right]\right)}^{-1}$.

Left multiplying both sides of Equation (22) by $S_{k}$ yields:

(24) $$D_{k}=M_{k}x_{k}+e_{k},$$

where $D_{k}=S_{k}\left[\begin{array}{c} {\bar{x}}_{k}\\ g\left(y_{k}\right) \end{array}\right]$, $M_{k}=S_{k}\left[\begin{array}{c} I_{n}\\ I_{n} \end{array}\right]$, $e_{k}=S_{k}\delta_{k}$.

The correntropy-based cost function is introduced as:

(25) $$J\left(x_{k}\right)=\frac{1}{2n}\sum_{i=1}^{2n}G_{\sigma }\left(d_{k,i}-m_{k,i}x_{k}\right),$$

where $d_{k,i}$ denotes the *i*-th component of $D_{k}$, $m_{k,i}$ denotes the *i*-th row of $M_{k}$, and *n* is the dimension of the state.

By the similar derivation in [55], the fixed-point iterative method would be adopted as:

(26) $${\hat{x}}_{k}^{\left(t\right)}={\left(M_{k}^{T}C_{k}^{\left(t-1\right)}M_{k}\right)}^{-1}M_{k}^{T}C_{k}^{\left(t-1\right)}D_{k},$$

where superscript (t) stands for the fixed-point iteration index, and $C_{k}^{\left(t-1\right)}=\left[\begin{array}{cc} C_{\chi ,k}^{\left(t-1\right)} & 0\\ 0 & C_{V,k}^{\left(t-1\right)} \end{array}\right]$, and the diagonal elements are defined in Equations (14) and (15). In fact, Equation (26) can be further written as Equation (7) (the detailed derivation can be seen in Appendix A).

### 3.2. Derivation of Information Matrix

After the iterative process stops, the estimation error information matrix ${\hat{\chi }}_{k}$ also needs to update. According to [56], the influence function (IF), defined as an *n* by 1 matrix, can be used to derive the error covariance matrix of an estimator under an assumed probability distribution, and there is a relationship between the influence function and the asymptotic covariance matrix of the estimation error ${\hat{P}}_{k}$ as follows:

(27) $${\hat{P}}_{k}=E\left[\mathrm{IF}\cdot {\mathrm{IF}}^{T}\right],$$

where E is the expectation operator. The detailed derivation can be seen in Appendix B. The IF can be computed as:

(28) $$\mathrm{IF}\left(e,m^{T},A_{0},T\right)=\frac{\varphi (e)}{E_{A_{0}}\left[\varphi^{\prime }\left(e\right)\right]}{\left[M_{k}^{T}M_{k}\right]}^{-1}m_{k,i}^{T},i=1,\ldots ,2n,$$

where $\varphi \left(e\right)=G_{\sigma }\left(e\right)e$, $E_{A_{0}}\left[\varphi^{\prime }\left(e\right)\right]$ is the expectation of the first derivative of φ(e) at distribution $A_{0}$, and $A_{0}$ is the target distribution of *e*. $m_{k,i}^{T}$ is the transposition of the *i*-th row of $M_{k}$, and $M_{k}$ is denoted in Equation (24). The detailed derivation can be seen in Appendix C. Thus the corresponding information matrix is written as:

(29) $${\hat{\chi }}_{k}=\frac{{\left\{E_{A_{0}}\left[\varphi^{\prime }\left(e\right)\right]\right\}}^{2}}{E_{A_{0}}\left[\varphi^{2}\left(e\right)\right]}M_{k}^{T}M_{k}.$$

Here we prove $E_{A_{0}}\left[\varphi^{\prime }\left(e\right)\right]$ and $E_{A_{0}}\left[\varphi^{2}\left(e\right)\right]$ as constants in Equations (A29) and (A30) of Appendix D, then substituting into Equation (29) yields:

(30) $${\hat{\chi }}_{k}=\frac{\sigma^{3}\left(\sigma^{2}+2\theta^{2}\right)\sqrt{\sigma^{2}+2\theta^{2}}}{\theta^{2}{\left(\sigma^{2}+\theta^{2}\right)}^{3}}M_{k}^{T}M_{k},$$

where σ is the kernel bandwidth, and θ is the variance of the distribution $A_{0}$. Note that the information matrix in Equation (30) can only hold in an asymptotic sense and can be used for the state with a high dimension. However, for the state with a small dimension, especially in the derivation of our algorithm, Equation (30) may not be a good choice. Equation (26) can be referred to as the iterative reweighted least squares (IRLS) method. Thus, assuming the iteration terminates at t=T, the final estimate is ${\hat{x}}_{k}^{\left(T\right)}$ and the associated covariance ${\hat{P}}_{k}$ can be obtained by:

(31) $${\hat{P}}_{k}={\left(M_{k}^{T}C_{k}^{\left(t-1\right)}M_{k}\right)}^{-1}.$$

Thus, the information matrix is computed as:

(32) $$\begin{array}{cc} {\hat{\chi }}_{k} & =M_{k}^{T}C_{k}^{\left(t-1\right)}M_{k}\\ \; & =S_{\chi ,k}^{T}C_{\chi ,k}^{\left(t-1\right)}S_{\chi ,k}+S_{V,k}^{T}C_{V,k}^{\left(t-1\right)}S_{V,k}\\ \; & ={\tilde{\chi }}_{k}^{\left(t-1\right)}+{\tilde{V}}_{k}^{\left(t-1\right)} \end{array}.$$

We can see that the information matrix in Equation (30) is equivalent to the least squares (LS) method, i.e., $M_{k}^{T}M_{k}$, multiplied by a constant scalar being smaller than one. The information matrix in Equation (32) implies that each row is multiplied by a different scalar depending on the residual error $e_{k,i}$ as in Equation (16). The outlier could be eliminated by multiplying a smaller scalar in calculating the information matrix. Thus compared to Equation (30), using Equation (32) may be relatively better in the small dimension case, especially in the proposed algorithm. Unless otherwise specified, the proposed algorithm uses the information matrix in Equation (32).

### 3.3. Robustness Analysis

According to [56], the influence function (IF) can also be used to measure quantitatively the robustness of an estimator, i.e., the infinitesimal robustness. If an estimator is infinitesimally robust, the corresponding IF needs to satisfy continuity and boundedness [57]. We show the IF satisfies continuity in Equation (28). Next, we will give the proof with respect to the boundedness of IF. According to Equation (28), $E_{A_{0}}\left[\varphi^{\prime }\left(e\right)\right]$ is a constant (please see in Appendix D) and $\left(M_{k}^{T}M_{k}\right)m_{k,i}^{T}$ is a known matrix, and we can see that the boundedness of IF depends on φ(e).

**Theorem** **1.** *The function φ(e) is bounded in the interval (−∞,+∞).*

**Proof** **of** **Theorem** **1.** (33) $\varphi \left(e\right)=e\times \exp\left(-\frac{e^{2}}{2\sigma^{2}}\right)=\frac{e}{\exp\left(\frac{e^{2}}{2\sigma^{2}}\right)},$

where *e* is the error and σ is the kernel bandwidth. Note that φ(e) is a continuous and odd function according to the boundedness theorem [58], thus it satisfies the boundedness in any closed intervals, i.e., if ∃τ>0, and φ(e) is bounded in the interval $\left[-\tau ,\tau \right]$. In the interval $\left[\tau ,+\infty \right]$, according to the L’ Hospital’s rule [59], we have:

(34) $$\lim_{e\to +\infty }\varphi \left(e\right)=\underset{e\to +\infty }{lim}\frac{e}{\exp\left(\frac{e^{2}}{2\sigma^{2}}\right)}=\underset{e\to +\infty }{lim}\frac{\sigma^{2}}{e\exp\left(\frac{e^{2}}{2\sigma^{2}}\right)}=0.$$

Consequently, φ(e) is bounded in the interval $\left[\tau ,+\infty \right)$, and it is bounded in the interval $\left(-\infty ,-\tau \right]$ due to the odd characteristic of the function φ(e). This completes the proof. The IF in Equation (28) satisfies continuity and boundedness, and the proposed algorithm can be regarded as infinitesimally robust. □

### 3.4. Convergence Analysis

In Section 3.1, we adopt a fixed-point iterative algorithm to obtain the posterior estimation in Equation (26). In fact, the convergence of the fixed-point iteration is impacted by the kernel bandwidth σ. According to the Banach Fixed-Point Theorem [60], the sufficient condition with respect to the choice of the kernel bandwidth to ensure the convergence of the fixed-point iteration is the same as in NMCIF [55,61].

Here we focus on discussing the convergence rate of the fixed-point iteration. For the sake of this discussion, Equation (26) can be written in an equivalent form as:

(35) $$x_{k}={\left(\sum_{i=1}^{2n}G_{\sigma }\left(e_{k,i}\right)m_{k,i}^{T}m_{k,i}\right)}^{-1}\sum_{i=1}^{2n}G_{\sigma }\left(e_{k,i}\right)d_{k,i}m_{k,i}^{T},$$

where $e_{k,i}$, $m_{k,i}$, and $d_{k,i}$ are defined as in Equation (24). Thus Equation (35) forms a fixed point equation as $x_{k}=f\left(x_{k}\right)$. Here we use $\nabla_{x_{k}}f\left(x_{k}\right)$ to denote the n×n Jacobian matrix of $f\left(x_{k}\right)$ with respect to $x_{k}$, that is:

(36) $$\nabla_{x_{k}}f\left(x_{k}\right)=\left[\frac{\partial }{\partial x_{k,1}}f\left(x_{k}\right)\ldots \frac{\partial }{\partial x_{k,n}}f\left(x_{k}\right)\right],$$

with

(37) $$\begin{array}{cc} \frac{\partial }{\partial x_{k,j}}f\left(x_{k}\right)= & -N_{\mathrm{mm}}^{-1}\left(\frac{1}{\sigma^{2}}\sum_{i=1}^{2n}\left[e_{k,i}m_{k,ij}G_{\sigma }\left(e_{k,i}\right)m_{k,i}^{T}m_{k,i}\right]\right)f\left(x_{k}\right)\\ \; & +N_{\mathrm{mm}}^{-1}\left(\frac{1}{\sigma^{2}}\sum_{i=1}^{2n}\left[e_{k,i}m_{k,ij}G_{\sigma }\left(e_{k,i}\right)m_{k,i}^{T}d_{k,i}\right]\right), \end{array}$$

where $N_{\mathrm{mm}}=\sum_{i=1}^{2n}G_{\sigma }\left(e_{k,i}\right)m_{k,i}^{T}m_{k,i}$ and $m_{k,ij}$ is the *j*-th element of $m_{k,i}$.

It is obvious that $G_{\sigma }\left(e_{k,i}\right)⩽1$, and according to the first sufficient condition ${∥f\left(x_{k}\right)∥}_{1}⩽\beta$ in [55] and Equation (37), we have:

(38) $$\begin{array}{cc} {∥\frac{\partial }{\partial x_{k,j}}f\left(x_{k}\right)∥}_{1}⩽ & {∥-N_{\mathrm{mm}}^{-1}∥}_{1}{∥\frac{1}{\sigma^{2}}\sum_{i=1}^{2n}\left[e_{k,i}m_{k,ij}G_{\sigma }\left(e_{k,i}\right)m_{k,i}^{T}m_{k,i}\right]∥}_{1}{∥f\left(x_{k}\right)∥}_{1}\\ \; & +{∥N_{\mathrm{mm}}^{-1}∥}_{1}{∥\frac{1}{\sigma^{2}}\sum_{i=1}^{2n}\left[e_{k,i}m_{k,ij}G_{\sigma }\left(e_{k,i}\right)m_{k,i}^{T}d_{k,i}\right]∥}_{1}\\ ⩽ & \frac{1}{\sigma^{2}}\beta {∥-N_{\mathrm{mm}}^{-1}∥}_{1}\sum_{i=1}^{2n}{∥e_{k,i}m_{k,ij}m_{k,i}^{T}m_{k,i}∥}_{1}\\ \; & +\frac{1}{\sigma^{2}}{∥N_{\mathrm{mm}}^{-1}∥}_{1}\sum_{i=1}^{2n}{∥e_{k,i}m_{k,ij}m_{k,i}^{T}d_{k,i}∥}_{1}, \end{array}$$

where ${∥.∥}_{1}$ denotes a 1-norm of a vector or an induced norm of a matrix. We can see since β, $N_{\mathrm{mm}}^{-1}$, $e_{k,i}$, $m_{k,i}$, and $d_{k,i}$ are bounded, it is evident that if we choose a large enough kernel bandwidth σ, the gradient vector is close to zero with a limited bounded 1-norm. Then the gradient at the optimal point is zero in practice and the algorithm is at least quadratically convergent. In particular, if σ→∞, we have $G_{\sigma }\left(e_{k,i}\right)\to 1$, then the fixed-point iteration method changes to the MMSE solution (i.e., the NIF in Section 2), which has a zero gradient vector in the optimal solution and converges in just one step. Consequently, when the kernel bandwidth σ decreases, the gradient of the fixed-point method at the optimal solution will increase, and the order of convergence will decrease from the super-linear to linear order of convergence [62]. Moreover, the robustness of the algorithm increases with a smaller kernel bandwidth.

By the above analysis, we can obtain that the smaller the kernel bandwidth, the more robust the algorithm is and the slower the convergence rate. However the kernel bandwidth has a low bound value to guarantee the convergence of the fixed-point method. On the other hand, when the kernel bandwidth becomes increasingly larger, the convergence rate increases, and the performance of the algorithm will behave like the corresponding NIF. Especially, if σ→∞, then $C_{k}\to I$, the proposed algorithm will reduce to the NIF and converge in just one step.

## 4. Neural Decoding Using Nonlinear Maximum Correntropy Information Filter

In this section, we apply the proposed nonlinear maximum correntropy information filter to decode the two-dimensional movement intention from high-dimensional neural observations, and make comparisons with the Kalman filter and multi-layer perception network.

### 4.1. Experiment and Data Collection

The BMI experiment paradigm of the rats’ two-lever discrimination task was designed and conducted in the Hong Kong University of Science and Technology. All animal handling procedures were approved by the Animal Care Committee of the Hong Kong University of Science and Technology. Two 16-channel microelectrode arrays were implanted in the primary motor cortex (M1) and medial prefrontal cortex (mPFC) respectively on the left hemisphere. The raw data of neural extracellular potential was recorded by a multi-channel acquisition processor (Plexon Inc, Dallas, Texas) with a 40-kHz frequency. The recorded potential was filtered by a 4-pole Butterworth high pass filter at 500 Hz and the action potentials were detected with an artificially set threshold (about 4 times of standard deviation of the filtered potential) in an online recording system. The spike trains were binned by a 100-ms time window without overlap for each channel. The count of spikes in the 100-ms window was assigned as firing rates.

The rats were trained to perform the two-lever discrimination task. In this task, the rats were required to distinguish two audio tones, which were randomly given by the computer at the trial start. The rats needed to use their right paw to press the corresponding lever and hold it for over 500 ms within a 6-second try-out time to get a water reward. With the high pitch (10 kHz), the rats needed to reach the high lever which was 10 cm high. While with the low tone (1.5 kHz), the rats turned to the low lever which wa spositioned 6 cm lower than the high lever. After successfully holding, the water was provided by a pump with a short feedback tone which had the same frequency as the start cue. The behavioral events and their timing information were recorded by the behavioral chamber (Lafayettee Instrument, Lafayettee, USA), and synchronized through the Plexon digital input with neural spike activity. There are totally 51 trials of a high lever press and 80 trials of a low lever press in rat A, as well as 72 trials of a high lever press and 128 trials of a low lever press in rat B.

**Figure 1:**
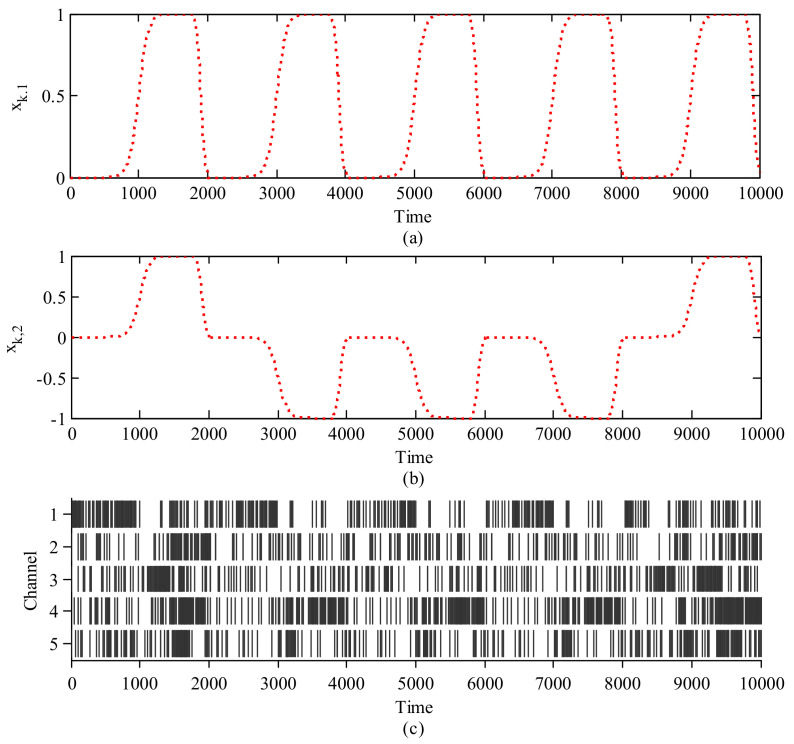
The 2−D behavioral states (**a**,**b**) and corresponding spike rasters in five channels of M1 (**c**).

To model the above procedure, we only consider the total successful trials, and the two-dimensional states are denoted as $x_{k}$. To label the behavioral data, all successful trials are segmented from start to 500 ms after the start, and from 500 ms before the press to the press onset. All segments are connected to represent the behavioral process and smoothed by a sigmoid function. After the feedback, the rats spend 200 ms returning rest state and it is smoothed by a sigmoid function as well. To label the behavioral data, the 500 ms before the start cue is set as the rest state with [0,0], and holding a high lever and low lever are set as [1,1] and [1,−1] respectively. We use the current time step of the neural firing rates as the 32-dimensional measurements, which are denoted as $y_{k}$ to decode the behavioral state. Figure 1c shows one segment of spike rasters over time in five channels. And the corresponding behavioral states in 2D are shown in Figure 1a,b respectively. Following the above design, the state equations and measurement equations can be written as Equations (1) and (8). The state transition F in Equation (1) is obtained by the least square approach, the nonlinear relationship g(·) in Equation (8) is approximated by a multi-layer perception network, and the mean and covariance of the process noise $q_{k}$ in Equation (1) and measurement noise $r_{k}$ in Equation (8) are estimated from the residual errors. Next according to the above model, we apply the proposed algorithm to estimate the two-dimensional states $x_{k}$ from the high-dimensional measurements $y_{k}$ and compare it with the KF and neural network decoding performance. Note that the neural network takes in the current neural firing rates with the past 400 ms firing rates history and outputs the 2-dimensional movement intention. The number of hidden PE is set as 10. The weights are initialized 20 times and trained by the steepest gradient-descent back propagation method, which minimizes the mean square error between the output and the ground truth movement. We use 60% of the training data for weight training and 40% for testing. We use the 2D-mean square error between the movement estimation and the ground truth as the criterion to evaluate the decoding performance.

### 4.2. Results

**Figure 2:**
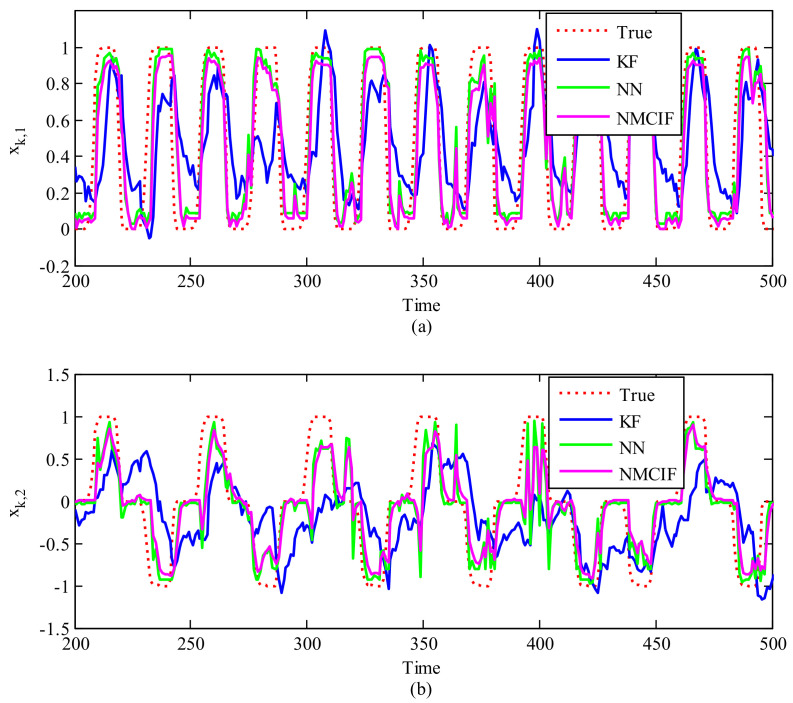
The reconstruction of 2−D movements over time by different models. The X−axis is the time, the Y−axis is the first (**a**) and the second (**b**) dimension of the movement. The red dotted curve is the ground truth, the blue solid curve is the Kalman filter, the magenta solid curve is the nonlinear maximum correntropy information filter, and the green solid curve is the neural network.

Figure 2a,b show a segment of decoded 2-D movements, $x_{k,1}$ and $x_{k,2}$ using KF, with NMCIF and NN presented for comparison. We can see that the curve of NMCIF (the magenta solid curve) is closer to the ground truth (the red dotted curve) than that of KF (the blue solid curve). This is because the proposed method adopts the nonlinear process of observation into the same dimension of the states instead of the linear model, which considers interactions among recorded neurons. Moreover, from the 210th time step to the 220th time step, the 320th time step to the 330th time step, and the 350th time step to the 400th time step in $x_{k,2}$ of Figure 2, we can see that the curve of NMCIF (the magenta solid curve) is smoother than that of NN (the green solid curve) due to the consideation of the time dynamic nature of the state. Table 1 shows the statistical 2D-MSEs of the algorithms between the true values and estimation values across 10 data segments with each rat. Please note that here for the NN, we use a MLP network to approximate the nonlinearity. We can also use RNN, where the results of RNN is 0.3555±0.0959 and 0.3984±0.0735. In terms of the averaged performance, the 2D-MSE of NMCIF in rat_A decreases by 57.62% and 6.91% compared with those of KF and NN, and the 2D-MSE of NMCIF in rat_B decreases by 25.89% and 5.17%. The results show that the proposed algorithm is superior to the KF and NN.

We then discuss convergence and sensitivity of the proposed algorithm with the presence of large deviated state initials.

**Figure 3:**
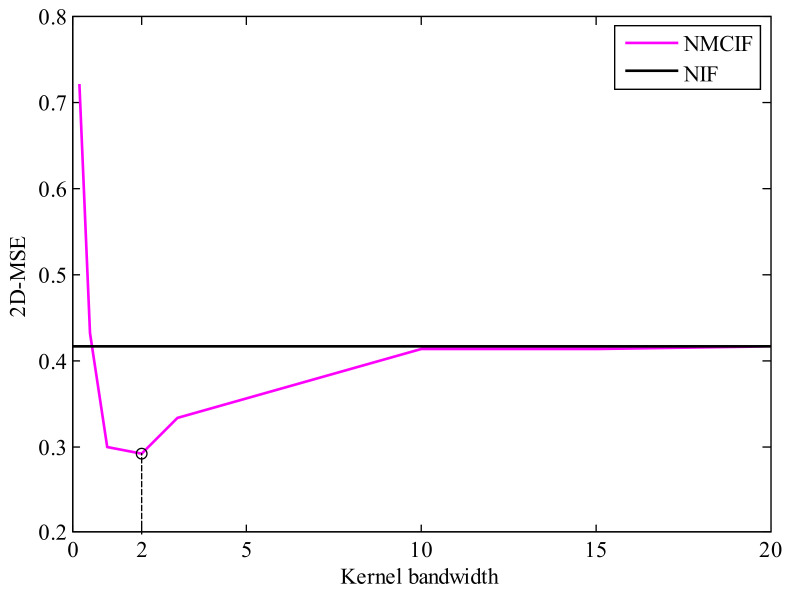
The relationship between the performance and kernel bandwidth.

**Figure 4:**
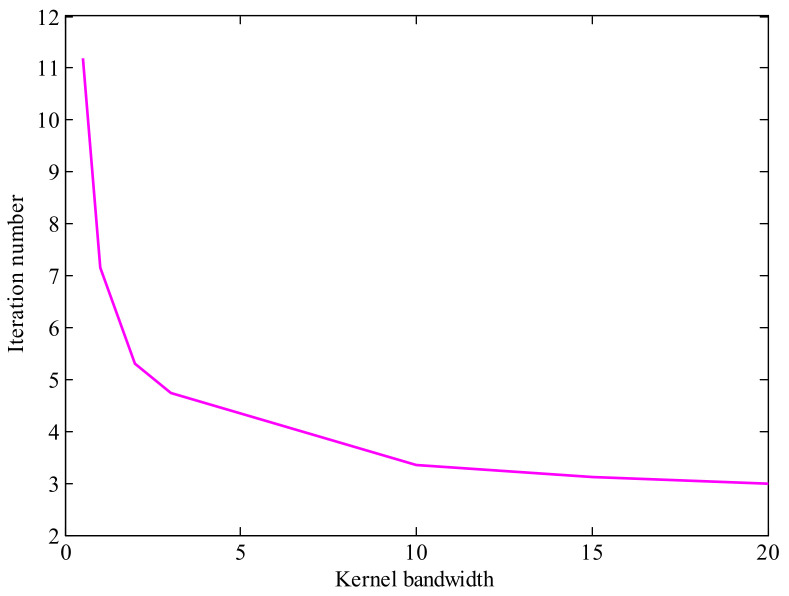
The relationship between the iteration number and kernel bandwidth.

From Section 3.4, we know that the kernel bandwidth plays a key role on the performance of NMCIF. Figure 3 shows the relationships between performance and the kernel bandwidth. We can see that if the kernel bandwidth is too small, the proposed algorithm plays a worse performance, even diverges. If the kernel bandwidth is too large, the performance of NMCIF (the magenta solid curve) is similar to the performance of NIF (the black solid curve). With a proper kernel bandwidth, the NMCIF outperforms the NIF. In particular, when the kernel bandwidth σ=2, the NMCIF has its best performance. Figure 4 shows the relationship between the fixed-point iteration number and the kernel bandwidth. As one can see, the larger the kernel bandwidth is, the less the iteration number.

**Figure 5:**
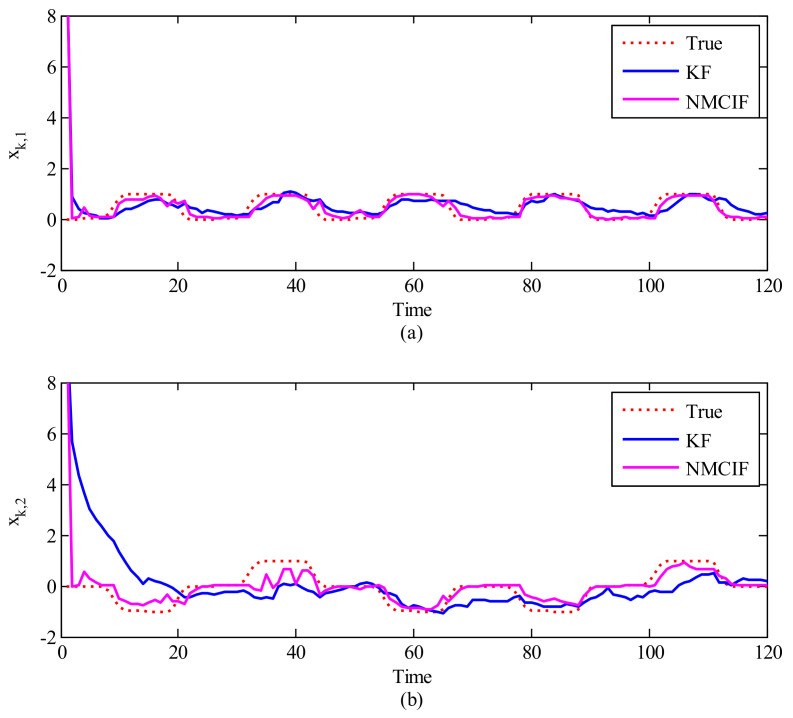
The reconstruction of 2−D movements with the initial value deviates greatly from the truth. The X−axis is the time, the Y−axis is the first (**a**) and the second (**b**) dimension of the movement. The red dotted curve is ground truth, the blue solid curve is the Kalman filter, and the magenta solid curve is the nonlinear maximum correntropy information filter.

Figure 5 shows the comparison between the KF and proposed NMCIF to exhibit the advantage of the information type filter when the initial condition is largely uncertain. The initial covariance matrix in KF is set to 1, but the initial information matrix in NMCIF is set to a small positive value ($10^{-6}$). Figure 5a,b show the estimation values of $x_{k,1}$ and $x_{k,2}$ in different algorithms compared with the ground truth on a segment of the data when the initial estimation value deviates greatly from the initial true value. As one can see, the NMCIF (the magenta solid curve) can converge quickly to the neighborhood of the ground truth (the red dotted curve), and KF (the blue solid curve) converges slowly. Table 2 summarizes the statistical decoding error over 10 segments of data with large deviated initial values. In terms of the averaged performance, the 2D-MSE of $x_{k}$ by NMCIF in rat_A and rat_B decrease by 33.84% and 26.00% respectively compared with that of KF. It is obvious that NMCIF is superior to KF when the initial estimation value deviates greatly from the initial true values.

**Figure 6:**
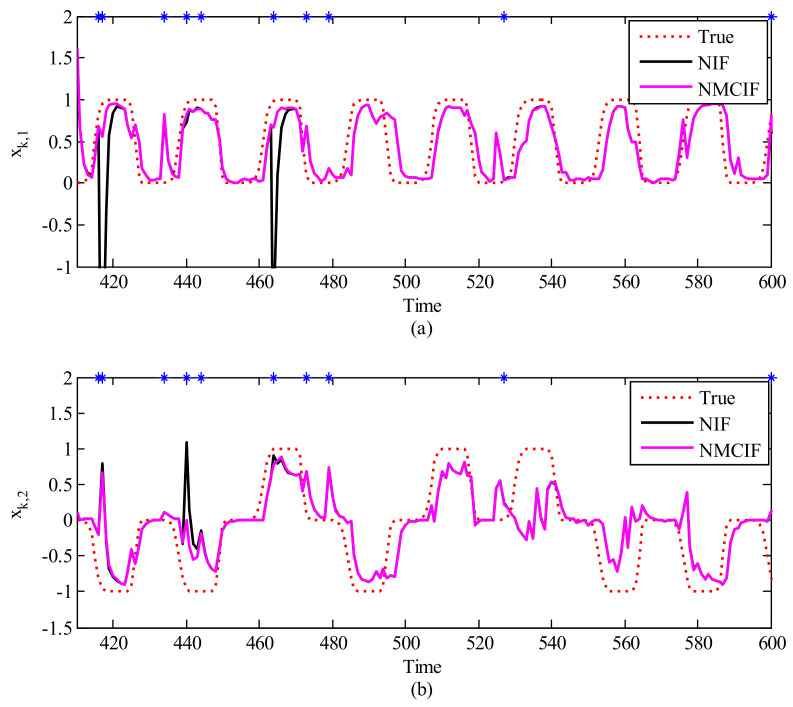
The reconstruction of 2−D movements affected by noisy observation. The X−axis is the time, The Y−axis is the first (**a**) and the second (**b**) dimension of the movement. The red dotted curve is ground truth, the black solid curve is the nonlinear information filter, and the magenta solid curve is the nonlinear maximum correntropy information filter.

As our algorithm demonstrates the best performance over KF and NN for neural decoding, here we further discuss the robustness of the proposed algorithm with the presence of large noise, which is generally seen in the online recorded neural activity [63]. Here 3.3% of time instances are added randomly with large noise, which corresponds to $r_{k}$ in Equation (8). Figure 6a,b show one segment of movement decoding using nonlinear information filter and the proposed method. In these figures, the red dotted curve denotes the ground truth and the blue asterisks denote the timing with large noise. The performance of NIF and NMCIF are presented for comparison. From the 410th time step to the 420th time step and the 460th time step to the 470th time step in Figure 6a and the 440th time step to the 450th time step in Figure 6b, we can see that the curve of NMCIF (the magenta solid curve) is smoother than that of NIF (the black solid curve) because of the usage of correntropy against the heavy-tailed non-Gaussian noise. The statistical 2D-MSEs of $x_{k}$ by NIF and NMCIF across 10 data segments are summarized in Table 3, respectively. Note that NMCIF_A denotes the NMCIF using the information matrix in Equation (30), and NMCIF_B denotes the NMCIF using the information matrix in Equation (32). In terms of the averaged performance, the 2D-MSE of NMCIF_B in rat_A and rat_B decrease by 29.54% and 8.97% respectively compared with that of NIF. Therefore, NMCIF exhibits better performance than NIF in non-Gaussian noise. The 2D-MSE of NMCIF_B is less than that of NMCIF_A, which indicates that the information matrix in Equation (32) may be relatively better in NMCIF.

## 5. Discussion and Conclusions

BMI records and decodes the neural signals to predict movement intents with the goal of helping better the lives of motor-impaired patients by restoring motor functions. As a widely-used decoding algorithm, KF is usually adopted to estimate the motor as the states from neural activity observations with the linear assumption and Gaussian noise. However, the nervous systems are nonlinear in general, and the observations are high-dimensional because recordings are collected from hundreds of neurons and could be very noisy, and the initial movement states of disabled patients cannot always be known. All these factors would result in the performance degradation of the KF. In this paper, we propose a nonlinear maximum correntropy information filter to derive a state in the filtering process from the noisy high-dimensional measurements. A nonlinear model is used to preprocess the high-dimensional neural observations into the same dimensionality as the states, which allows the connectivity among neurons to be considered. The correntropy criterion is adopted to address the presence of the non-Gaussian noise. The information matrix of the state is derived with less sensitivity to the state’s initial conditions. We provide an analysis of the convergence condition and robustness. The proposed algorithm is applied to decode the movements from real neural data collected from rats performing a two-lever discrimination task. The 2D reconstruction error (2D-MSE) of NMCIF in rat_A decreases by 57.62% and 6.91% compared with those of KF and NN, and the 2D-MSE of NMCIF in rat_B decreases by 25.89% and 5.17%. The results demonstrate that the nonlinear model considering the connection of neural activities contributes to better estimation performance. When the initial estimation value deviates greatly from the initial true value, the 2D-MSEs of NMCIF in rat_A and rat_B decrease by 33.84% and 26.00% respectively compared with those of KF. These exhibit the superiority of the information matrix type filter. Moreover, the 2D-MSEs of NMCIF in rat_A and rat_B decrease by 29.54% and 8.97% respectively compared with those of NIF in the presence of heavy-tailed non-Gaussian noise showing the proposed algorithm’s superiority to the NIF. These results confirm the effectiveness of the proposed algorithm, which potentially improves the decoding performance for BMI. In our study, the NN used as the nonlinear approximator is not limited to MLP but can also be RNN etc., as RNN is usually used in speech recognition and natural language processing. In the area of motor intention estimation, it has been recently used to classify the letter shapes using invasive brain signals [37]. However, the training of MLP is much simpler than the backpropagation through time in RNN. Our method follows the combination of the neural network and simple state-observation model. It inherits a simple explanation on state dynamics and observation model, which is correspondingly the neural tuning property and neural connectivity. On the other hand, the neural tuning property generally changes over time [64,65], the performance of static decoder may decrease if we use the fixed observation model. Thus considering the adaptation in the neural system, the neural network in our method allows the introduction of adaptive nonlinear models [66,67] to replace the stationary observation model. Our study currently uses invasive recordings on rat’s single neuron spikes. The rat is an ideal subject for estimating motor intention and is widely used in many papers [68,69,70], and the Kalman filter has been used in rats [39], monkeys [40], and human subjects [71]. We plan to utilize our algorithm on non-human primates and patients in the future. As more electrodes could be implanted in such subjects, the recorded neural signals would build higher dimensional observations. We would expect the superiority of the proposed algorithm to be clearer on these data.

## Figures and Tables

**Table 1:** Statistical decoding performances across 10 segments of test data with Different rats.

Method	2D-MSE of $x_{k}$ in Rat_A	2D-MSE of $x_{k}$ in Rat_B
KF	0.5783 ± 0.1074	0.5558 ± 0.0653
NN	0.2633 ± 0.0787	0.4119 ± 0.0588
NMCIF	0.2451 ± 0.0684	0.3906 ± 0.0491

**Table 2:** Statistical decoding performances across 10 segments of test data with large deviated initial values.

Method	2D-MSE of $x_{k}$ in Rat_A	2D-MSE of $x_{k}$ in Rat_B
KF	2.8686 ± 0.2112	1.8142 ± 0.1218
NMCIF	1.8978 ± 0.0661	1.3425 ± 0.0477

**Table 3:** Statistical decoding performances across 10 segments of test data affected by non-Gaussian noise.

Method	2D-MSE of $x_{k}$ in Rat_A	2D-MSE of $x_{k}$ in Rat_B
NIF	0.4113 ± 0.1165	0.4962 ± 0.0456
NMCIF_A	0.2933 ± 0.0630	0.4548 ± 0.0453
NMCIF_B	0.2898 ± 0.0637	0.4517 ± 0.0437

## Data Availability

Data sharing not applicable.

## Appendix A. Derivation Process from Equation (26) to Equation (7)

In this part, we derive Equation (26) to Equation (7). According to Equations (24) and (26), the following equations can be:

(A1) $$M_{k}=S_{k}\left[\begin{array}{c} I_{n}\\ I_{n} \end{array}\right]=\left[\begin{array}{cc} S_{\chi ,k} & 0\\ 0 & S_{V,k} \end{array}\right]\left[\begin{array}{c} I_{n}\\ I_{n} \end{array}\right]=\left[\begin{array}{c} S_{\chi ,k}\\ S_{V,k} \end{array}\right],$$

(A2) $$C_{k}^{\left(t-1\right)}=\left[\begin{array}{cc} C_{\chi ,k}^{\left(t-1\right)} & 0\\ 0 & C_{V,k}^{\left(t-1\right)} \end{array}\right],$$

(A3) $$D_{k}=S_{k}\left[\begin{array}{c} {\bar{x}}_{k}\\ g\left(y_{k}\right) \end{array}\right]=\left[\begin{array}{c} S_{\chi ,k}{\bar{x}}_{k}\\ S_{V,k}g\left(y_{k}\right) \end{array}\right],$$

(A4) $${\left(M_{k}^{T}C_{k}^{\left(t-1\right)}M_{k}\right)}^{-1}={\left[S_{\chi ,k}^{T}C_{\chi ,k}^{\left(t-1\right)}S_{\chi ,k}+S_{V,k}^{T}C_{V,k}^{\left(t-1\right)}S_{V,k}\right]}^{-1}.$$

Using the following matrix inversion lemma:

(A5) $${\left(A+BD^{-1}C\right)}^{-1}=A^{-1}-A^{-1}B{\left(D+CA^{-1}B\right)}^{-1}CA^{-1},$$

(A6) $$\begin{array}{c} S_{\chi ,k}^{T}C_{\chi ,k}^{\left(t-1\right)}S_{\chi ,k}\to A,I_{n}\to B,\\ I_{n}\to C,S_{V,k}^{T}C_{V,k}^{\left(t-1\right)}S_{V,k}\to D^{-1}. \end{array}$$

Equation (A4) can also be written as:

(A7) $$\begin{array}{cc} {\left(M_{k}^{T}C_{k}^{\left(t-1\right)}M_{k}\right)}^{-1}= & S_{\chi ,k}^{-1}{\left(C_{\chi ,k}^{\left(t-1\right)}\right)}^{-1}{\left(S_{\chi ,k}^{T}\right)}^{-1}-S_{\chi ,k}^{-1}{\left(C_{\chi ,k}^{\left(t-1\right)}\right)}^{-1}{\left(S_{\chi ,k}^{T}\right)}^{-1}\\ \; & \times {\left(S_{V,k}^{-1}{\left(C_{V,k}^{\left(t-1\right)}\right)}^{-1}{\left(S_{V,k}^{T}\right)}^{-1}+S_{\chi ,k}^{-1}{\left(C_{\chi ,k}^{\left(t-1\right)}\right)}^{-1}{\left(S_{\chi ,k}^{T}\right)}^{-1}\right)}^{-1}\\ \; & \times S_{\chi ,k}^{-1}{\left(C_{\chi ,k}^{\left(t-1\right)}\right)}^{-1}{\left(S_{\chi ,k}^{T}\right)}^{-1}. \end{array}$$

By Equations (A1)∼ (A3), we have:

(A8) $$M_{k}^{T}C_{k}^{\left(t-1\right)}D_{k}=S_{\chi ,k}^{T}C_{\chi ,k}^{\left(t-1\right)}S_{\chi ,k}{\bar{x}}_{k}+S_{V,k}^{T}C_{V,k}^{\left(t-1\right)}S_{V,k}g\left(y_{k}\right).$$

By substituting Equations (A7) and (A8) into Equation (26), we have Equation (7).

## Appendix B. The Relationship of the Influence Function and Asymptotic Covariance Matrix

Following [57], we derive the relationship of the IF to the asymptotic covariance matrix of the estimation error vector. Using a form of Taylor expansion involving the derivative of the functional to express the estimator T(A) yields:

(A9) $$T\left(A\right)=T\left(A_{0}\right)+T^{\prime }\left(A-A_{0}\right)+rem\left(A-A_{0}\right),$$

where $rem(A-A_{0})$ is a remainder term. According to the von Mises expansion and assuming the IF exists, Equation (A9) can be written as:

(A10) $$T\left(A\right)=T\left(A_{0}\right)+\int \mathrm{IF}\left(e,A_{0}\right)d\left(A-A_{0}\right)+rem\left(A-A_{0}\right).$$

Since the Fisher consistency at $A_{0}$, obtained by $\int \mathrm{IF}\left(e,A_{0}\right)d\left(A_{0}\right)=0$, we have:

(A11) $$T\left(A\right)=T\left(A_{0}\right)+\int \mathrm{IF}(e,A_{0})dA+rem\left(A-A_{0}\right).$$

For $A=F_{m}\left(e\right)$ with $F_{m}\left(e\right)$ being the empirical distribution function, obtained by $F_{m}\left(e\right)=\left(1/m\right)\sum_{i=1}^{m}u(e-e_{i})$ with u(·) being the unit step function, the integral term in Equation (A11) can be written in linear term as:

(A12) $$\int \mathrm{IF}\left(e,A_{0}\right)dF_{m}=\frac{1}{m}\sum_{i=1}^{m}\mathrm{IF}(e_{i},A_{0}).$$

Then, we have:

(A13) $$\sqrt{m}\left(T\left(F_{m}\right)-T\left(A_{0}\right)\right)=\frac{1}{\sqrt{m}}\sum_{i=1}^{m}{\mathrm{IF}}_{i}\left(e,A_{0}\right)+\sqrt{m}rem\left(F_{m}-A_{0}\right).$$

If the remainder term converges in probability to zero, then by virtue of the central limit theorem and Slutsky’s lemma, the probability distribution of $\sqrt{m}\left(T\left(F_{m}\right)-T\left(A_{0}\right)\right)$ tends to $N(0,\Lambda_{k})$, with an asymptotic covariance matrix given by:

(A14) $${\hat{P}}_{k}=E\left[\mathrm{IF}\cdot {\mathrm{IF}}^{T}\right].$$

## Appendix C. The Derivation of Influence Function

For the sake of this discuss, we omit the time step *k*. From Equation (24), we can obtain the residual vector e=D−Mx. Consider a ε-contamination model $A=\left(1-\varepsilon \right)A_{0}+\varepsilon \Delta e$, where $A_{0}$ is the target distribution, Δe is the probability mass at *e*, and ε is the contaminating ratio, and define the cumulative probability distribution of the residual vector as $A_{0}\left(e\right)$. In general, the state estimate based on correntropy can be obtained by solving Equation (26), which can be written in an equivalent form as:

(A15) $$\sum_{i=1}^{2n}\lambda (e_{i},m_{i}^{T},x)=\sum_{i=1}^{2n}G_{\sigma }\left(e_{i}\right)e_{i}m_{i}^{T}=0.$$

Let T(A) be an estimator based on correntropy (i.e., state estimate ${\hat{x}}_{k}$ at the distribution *A*), which is written in a functional form of *A*. If each $\lambda (e_{i},m_{i}^{T},x)$ in Equation (A15) is left-multiplied by probability mass $\frac{1}{2n}$, and from the Glivenko–Cantelli theorem [72], Equation (A15) asymptotically tends to:

(A16) $$\int \lambda \left(e,m^{T},T\left(A\right)\right)dA=0,$$

where *e* and $m^{T}$ are generalizations of $e_{i}$ and $m_{i}^{T}$. According to [56], the asymptotic IF of T(A) is defined as:

(A17) $$\mathrm{IF}\left(e,m^{T},A_{0},T\right)={\left.\frac{\partial T(A)}{\partial \varepsilon }\right|}_{\varepsilon =0}=\lim_{\Delta \varepsilon \to 0}\frac{T\left(\left(1-\varepsilon \right)A_{0}+\varepsilon \Delta e\right)-T\left(A_{0}\right)}{\varepsilon }.$$

To derive the expression of IF, we substitute the ε-contamination model $A=\left(1-\varepsilon \right)A_{0}+\varepsilon \Delta e$ into Equation (A16) to yield:

(A18) $$\begin{array}{cc} & \int \lambda \left(e,m^{T},T\left(A\right)\right)dA\\ = & \int \lambda \left(e,m^{T},T\left(A\right)\right)d\left(\left(1-\varepsilon \right)A_{0}+\varepsilon \Delta e\right)\\ = & \int \lambda \left(e,m^{T},T\left(A\right)\right)dA_{0}+\varepsilon \int \lambda \left(e,m^{T},T\left(A\right)\right)d\left(\Delta e-A_{0}\right)=0. \end{array}$$

Taking the differentiation of Equation (A18) with respect to ε yields:

(A19) $$\begin{array}{cc} \; & \frac{\partial }{\partial \varepsilon }\int \lambda \left(e,m^{T},T\left(A\right)\right)dA_{0}+\int \lambda \left(e,m^{T},T\left(A\right)\right)d\left(\Delta e-A_{0}\right)\\ \; & +\varepsilon \frac{d}{d\varepsilon }\int \lambda \left(e,m^{T},T\left(A\right)\right)d\left(\Delta e-A_{0}\right)=0. \end{array}$$

Calculating Equation (A19) at ε=0, and assuming T(A) at the distribution $A_{0}$ follows the Fisher consistency and regularity conditions, obtained by $\int \lambda \left(e,m^{T},T\left(A_{0}\right)\right)dA_{0}=0$, and utilizing the interchangeability of differentiation and integration theorem, we have:

(A20) $$\int {\left.\frac{\partial }{\partial \varepsilon }\lambda \left(e,m^{T},T\left(A\right)\right)dA_{0}\right|}_{\varepsilon =0}+\int {\left.\lambda \left(e,m^{T},T\left(A\right)\right)d\left(\Delta e\right)\right|}_{\varepsilon =0}=0.$$

Utilizing the shifting property of the Δe yields:

(A21) $$\int {\left.\frac{\partial }{\partial \varepsilon }\lambda \left(e,m^{T},T\left(A\right)\right)dA_{0}\right|}_{\varepsilon =0}+\lambda \left(e,m^{T},T\left(A_{0}\right)\right)=0.$$

Assuming $\lambda \left(\cdot \right)$ is continuous and measurable and $\lambda^{\prime }\left(\cdot \right)$ is measurable, we have:

(A22) $$\begin{array}{cc} \; & \int {\left.\frac{\partial }{\partial \varepsilon }\lambda \left(e,m^{T},T\left(A\right)\right)dA_{0}\right|}_{\varepsilon =0}+\lambda \left(e,m^{T},T\left(A_{0}\right)\right)\\ = & \int {\left.\frac{\partial \lambda \left(e,m^{T},T\left(A\right)\right)}{\partial T(A)}\right|}_{T(A_{0})}\cdot {\left.\frac{\partial T(A)}{\partial \varepsilon }\right|}_{\varepsilon =0}dA_{0}+\lambda \left(e,m^{T},T\left(A_{0}\right)\right)=0. \end{array}$$

Since T(A) is ${\hat{x}}_{k}$ at the distribution *A*, we have:

(A23) $$\begin{array}{cc} \mathrm{IF}(e,m^{T},A_{0},T) & ={\left.\frac{\partial T(A)}{\partial \varepsilon }\right|}_{\varepsilon =0}\\ \; & =-{\left[{\int \left.\frac{\partial \lambda \left(e,m^{T},T\left(A\right)\right)}{\partial T(A)}\right|}_{T(A_{0})}dA_{0}\right]}^{-1}\lambda \left(e,m^{T},T\left(A_{0}\right)\right)\\ \; & =-{\left[{\int \left.\frac{\partial \lambda \left(e,m^{T},x\right)}{\partial x}\right|}_{T(A_{0})}dA_{0}\right]}^{-1}\lambda \left(e,m^{T},T\left(A_{0}\right)\right). \end{array}$$

Taking the differentiation of $\lambda \left(e,m^{T},x\right)$ in Equation (A23) with respect to x yields:

(A24) $$\frac{\partial \lambda (e,m^{T},x_{k})}{\partial x_{k}}=\frac{\partial \varphi (e)}{\partial x_{k}}m^{T}=\frac{\partial \varphi (e)}{\partial e}\frac{\partial e}{\partial x_{k}}m^{T}=-\varphi^{\prime }\left(e\right)m^{T}m.$$

where $\varphi \left(e\right)=G_{\sigma }\left(e\right)e$.

By substituting Equations (24), (A15), and (A24) into Equation (A23) yields:

(A25) $$\mathrm{IF}\left(e,m^{T},A_{0},T\right)={\left[\int \varphi^{\prime }\left(e\right)m^{T}mdA_{0}\right]}_{T(A_{0})}^{-1}\varphi \left(e\right)m^{T}.$$

The integral in Equation (A25) can be regarded as computing the ensemble average of the function of the term $\varphi^{\prime }\left(e\right)$ for every $\left(m_{i}^{T}m_{i}\right),i=1,\ldots ,2n$, and it can be factored out as:

(A26) $$\int \varphi^{\prime }\left(e\right)m^{T}mdA_{0}=E_{A_{0}}\left[\varphi^{\prime }\left(e\right)\right]M^{T}M.$$

Then, we have:

(A27) $$\mathrm{IF}\left(e,m^{T},A_{0},T\right)=\frac{\varphi (e)}{E_{A_{0}}\left[\varphi^{\prime }\left(e\right)\right]}{\left[M^{T}M\right]}^{-1}m^{T}.$$

Since $E_{A_{0}}\left[\varphi^{\prime }\left(e\right)\right]$ is a constant (please see in Appendix D) and $\left(M^{T}M\right)m^{T}$ is a known matrix, the IF in Equation (A27) is proportional to φ(e). IF can evaluate the influence of each elements of the vector $e_{k}$ in Equation (24), i.e.,

(A28) $$\mathrm{IF}\left(e,m^{T},A_{0},T\right)=\frac{\varphi (d_{k,i}-m_{k,i}x_{k})}{E_{A_{0}}\left[\varphi^{\prime }\left(e\right)\right]}{\left[M_{k}^{T}M_{k}\right]}^{-1}m_{k,i}^{T},i=1,\ldots ,2n.$$

## Appendix D. The Solution of $E_{A_{0}}\left[\varphi^{\prime }\left(e\right)\right]$ and $E_{A_{0}}\left[\varphi^{2}\left(e\right)\right]$

Assume the distribution $A_{0}$ obeying a Gaussian distribution with zero mean and variance θ, we have:

(A29) $$\begin{array}{c} E_{A_{0}}\left[\varphi^{\prime }\left(e\right)\right]=\int_{-\infty }^{+\infty }\varphi^{\prime }\left(e\right)dA_{0}\\ =\int_{-\infty }^{+\infty }\left(\exp\left(-\frac{e^{2}}{2\sigma^{2}}\right)-\frac{e^{2}}{\sigma^{2}}\exp\left(-\frac{e^{2}}{2\sigma^{2}}\right)\right)dA_{0}\\ =\int_{-\infty }^{+\infty }\exp\left(-\frac{e^{2}}{2\sigma^{2}}\right)\frac{1}{\sqrt{2\pi }\theta }\exp\left(-\frac{e^{2}}{2\theta^{2}}\right)de\\ \;-\int_{-\infty }^{+\infty }\frac{e^{2}}{\sigma^{2}}\exp\left(-\frac{e^{2}}{2\sigma^{2}}\right)\frac{1}{\sqrt{2\pi }\theta }\exp\left(-\frac{e^{2}}{2\theta^{2}}\right)de\\ =\frac{1}{\sqrt{2\pi }\theta }\int_{-\infty }^{+\infty }\exp\left(-\frac{e^{2}}{2\frac{\sigma^{2}\theta^{2}}{\sigma^{2}+\theta^{2}}}\right)de-\frac{1}{\sqrt{2\pi }\theta \sigma^{2}}\int_{-\infty }^{+\infty }e^{2}\exp\left(-\frac{e^{2}}{2\frac{\sigma^{2}\theta^{2}}{\sigma^{2}+\theta^{2}}}\right)de\\ =\frac{\sigma }{\sqrt{\sigma^{2}+\theta^{2}}}+\frac{1}{\sqrt{2\pi }\theta \sigma^{2}}\frac{\sigma^{2}\theta^{2}}{\sigma^{2}+\theta^{2}}\int_{-\infty }^{+\infty }ed\exp\left(-\frac{e^{2}}{2\frac{\sigma^{2}\theta^{2}}{\sigma^{2}+\theta^{2}}}\right)\\ =\frac{\sigma }{\sqrt{\sigma^{2}+\theta^{2}}}+\frac{\theta }{\sqrt{2\pi }\left(\sigma^{2}+\theta^{2}\right)}\left({\left.e\cdot \exp\left(-\frac{e^{2}}{2\frac{\sigma^{2}\theta^{2}}{\sigma^{2}+\theta^{2}}}\right)\right|}_{-\infty }^{+\infty }-\int_{-\infty }^{+\infty }\exp\left(-\frac{e^{2}}{2\frac{\sigma^{2}\theta^{2}}{\sigma^{2}+\theta^{2}}}\right)de\right)\\ =\frac{\sigma }{\sqrt{\sigma^{2}+\theta^{2}}}+\frac{\theta }{\sqrt{2\pi }\left(\sigma^{2}+\theta^{2}\right)}\left(-\frac{\sqrt{2\pi }\sigma \theta }{\sqrt{\sigma^{2}+\theta^{2}}}\right)\\ =\frac{\sigma^{3}}{\left(\sigma^{2}+\theta^{2}\right)\sqrt{\sigma^{2}+\theta^{2}}} \end{array}.$$

(A30) $$\begin{array}{c} E_{A_{0}}\left[\varphi^{2}\left(e\right)\right]=\int_{-\infty }^{+\infty }\varphi^{2}\left(e\right)dA_{0}\\ =\int_{-\infty }^{+\infty }\left(e^{2}\exp\left(-\frac{e^{2}}{\sigma^{2}}\right)\right)dA_{0}\\ =\int_{-\infty }^{+\infty }e^{2}\exp\left(-\frac{e^{2}}{\sigma^{2}}\right)\frac{1}{\sqrt{2\pi }\theta }\exp\left(-\frac{e^{2}}{2\theta^{2}}\right)de\\ =\frac{1}{\sqrt{2\pi }\theta }\int_{-\infty }^{+\infty }e^{2}\exp\left(-\frac{e^{2}}{2\frac{\sigma^{2}\theta^{2}}{\sigma^{2}+2\theta^{2}}}\right)de\\ =-\frac{1}{\sqrt{2\pi }\theta }\frac{\sigma^{2}\theta^{2}}{\sigma^{2}+2\theta^{2}}\int_{-\infty }^{+\infty }ed\exp\left(-\frac{e^{2}}{2\frac{\sigma^{2}\theta^{2}}{\sigma^{2}+2\theta^{2}}}\right)\\ =-\frac{\sigma^{2}\theta }{\sqrt{2\pi }\left(\sigma^{2}+2\theta^{2}\right)}\left({\left.e\cdot \exp\left(-\frac{e^{2}}{2\frac{\sigma^{2}\theta^{2}}{\sigma^{2}+2\theta^{2}}}\right)\right|}_{-\infty }^{+\infty }-\int_{-\infty }^{+\infty }\exp\left(-\frac{e^{2}}{2\frac{\sigma^{2}\theta^{2}}{\sigma^{2}+2\theta^{2}}}\right)de\right)\\ =-\frac{\sigma^{2}\theta }{\sqrt{2\pi }\left(\sigma^{2}+2\theta^{2}\right)}\left(-\frac{\sqrt{2\pi }\sigma \theta }{\sqrt{\sigma^{2}+2\theta^{2}}}\right)\\ =\frac{\sigma^{3}\theta^{2}}{\left(\sigma^{2}+2\theta^{2}\right)\sqrt{\sigma^{2}+2\theta^{2}}} \end{array}.$$

## References

[1] Lebedev M.A., Nicolelis M.A.L. (2006). Brain–machine interfaces: Past, present and future. Trends Neurosci..

[2] Taylor D.M., Tillery S.I.H., Schwartz A.B. (2002). Direct Cortical Control of 3D Neuroprosthetic Devices. Science.

[3] O’Doherty J.E., Lebedev M.A., Hanson T.L., Fitzsimmons N.A., Nicolelis M.A.L. (2009). A brain-machine interface instructed by direct intracorticalmicrostimulation. Front. Integr. Neurosci..

[4] Orsborn A.L., Moorman H.G., Overduin S.A., Shanechi M.M., Dimitrov D.F., Carmena J.M. (2014). Closed-Loop Decoder Adaptation Shapes Neural Plasticity for Skillful Neuroprosthetic Control. Neuron.

[5] Vyas S., Even-Chen N., Stavisky S.D., Ryu S.I., Nuyujukian P., Shenoy K.V. (2018). Neural Population Dynamics Underlying Motor Learning Transfer. Neuron.

[6] Wolpaw J.R., Birbaumer N., Heetderks W.J., McFarland D.J., Peckham P.H., Schalk G., Donchin E., Quatrano L.A., Robinson C.J., Vaughan T.M. (2000). Brain-computer interface technology: A review of the first international meeting. IEEE Trans. Rehabil. Eng..

[7] Nicolelis M.A.L. (2003). Brain-machine interfaces to restore motor function and probe neural circuits. Nat. Rev. Neurosci..

[8] Churchland M.M., Cunningham J.P., Kaufman M.T., Foster J.D., Nuyujukian P., Ryu S.I., Shenoy K.V. (2012). Neural population dynamics during reaching. Nature.

[9] Musallam S., Corneil B.D., Greger B., Scherberger H., Andersen R.A. (2004). Cognitive Control Signals for Neural Prosthetics. Science.

[10] Velliste M., Perel S., Spalding M.C., Whitford A.S., Schwartz A.B. (2008). Cortical control of a prosthetic arm for self-feeding. Nature.

[11] Hochberg L.R., Serruya M.D., Friehs G.M., Mukand J.A., Saleh M., Caplan A.H., Branner A., Chen D., Penn R.D., Donoghue J.P. (2006). Neuronal ensemble control of prosthetic devices by a human with tetraplegia. Nature.

[12] Hochberg L.R., Bacher D., Jarosiewicz B., Masse N.Y., Simeral J.D., Vogel J., Haddadin S., Liu J., Cash S.S., van der Smagt P. (2012). Reach and grasp by people with tetraplegia using a neurally controlled robotic arm. Nature.

[13] Truccolo W., Friehs G.M., Donoghue J.P., Hochberg L.R. (2008). Primary Motor Cortex Tuning to Intended Movement Kinematics in Humans with Tetraplegia. J. Neurosci..

[14] Moritz C.T., Perlmutter S.I., Fetz E.E. (2008). Direct control of paralysed muscles by cortical neurons. Nature.

[15] Collinger J.L., Wodlinger B., Downey J.E., Wang W., Tyler-Kabara E.C., Weber D.J., McMorland A.J.C., Velliste M., Boninger M.L., Schwartz A.B. (2013). High-performance neuroprosthetic control by an individual with tetraplegia. Lancet.

[16] Gilja V., Pandarinath C., Blabe C.H., Nuyujukian P., Simeral J.D., Sarma A.A., Sorice B.L., Perge J.A., Jarosiewicz B., Hochberg L.R. (2015). Clinical translation of a high-performance neural prosthesis. Nat. Med..

[17] Bouton C.E., Shaikhouni A., Annetta N.V., Bockbrader M.A., Friedenberg D.A., Nielson D.M., Sharma G., Sederberg P.B., Glenn B.C., Mysiw W.J. (2016). Restoring cortical control of functional movement in a human with quadriplegia. Nat. Med..

[18] Ajiboye A.B., Willett F.R., Young D.R., Memberg W.D., Murphy B.A., Miller J.P., Walter B.L., Sweet J.A., Hoyen H.A., Keith M.W. (2017). Restoration of reaching and grasping movements through brain-controlled muscle stimulation in a person with tetraplegia: A proof-of-concept demonstration. Lancet.

[19] Brandman D.M., Hosman T., Saab J., Burkhart M.C., Shanahan B.E., Ciancibello J.G., Sarma A.A., Milstein D.J., Vargas-Irwin C.E., Franco B. (2018). Rapid calibration of an intracortical brain–computer interface for people with tetraplegia. J. Neural Eng..

[20] Gilja V., Nuyujukian P., Chestek C.A., Cunningham J.P., Yu B.M., Fan J.M., Churchland M.M., Kaufman M.T., Kao J.C., Ryu S.I. (2012). A high-performance neural prosthesis enabled by control algorithm design. Nat. Neurosci..

[21] Malik W.Q., Truccolo W., Brown E.N., Hochberg L.R. (2011). Efficient Decoding With Steady-State Kalman Filter in Neural Interface Systems. IEEE Trans. Neural Syst. Rehabil. Eng..

[22] Homer M.L., Perge J.A., Black M.J., Harrison M.T., Cash S.S., Hochberg L.R. (2014). Adaptive Offset Correction for Intracortical Brain-Computer Interfaces. IEEE Trans. Neural Syst. Rehabil. Eng..

[23] Wu W., Gao Y., Bienenstock E., Donoghue J.P., Black M.J. (2006). Bayesian Population Decoding of Motor Cortical Activity Using a Kalman Filter. Neural Comput..

[24] Thayer J.F. (2006). On the importance of inhibition: Central and peripheral manifestations of nonlinear inhibitory processes in neural systems. Dose-Response.

[25] Yang Y., Dewald J.P.A., van der Helm F.C.T., Schouten A.C. (2018). Unveiling neural coupling within the sensorimotor system: Directionality and nonlinearity. Eur. J. Neurosci..

[26] Anderson B., Moore J. (1979). Optimal Filtering.

[27] Julier S., Uhlmann J., Durrant-Whyte H.F. (2000). A new method for the nonlinear transformation of means and covariances in filters and estimators. IEEE Trans. Autom. Control..

[28] Li Z., O’Doherty J.E., Hanson T.L., Lebedev M.A., Henriquez C.S., Nicolelis M.A.L. (2009). Unscented Kalman Filter for Brain-Machine Interfaces. PLoS ONE.

[29] Arasaratnam I., Haykin S. (2009). Cubature Kalman filters. IEEE Trans. Autom. Control..

[30] Truccolp W., Eden U.T., Fellows M.R., Donoghue J.P., Brown E.N. (2005). A Point Process Framework for Relating Neural Spiking Activity to Spiking History, Neural Ensemble, and Extrinsic Covariate Effects. J. Neurophysiol..

[31] Qian C., Sun X., Zhang S., Xing D., Li H., Zheng X., Pan G., Wang Y. (2018). Nonlinear Modeling of Neural Interaction for Spike Prediction Using the Staged Point-Process Model. Neural Comput..

[32] Qian C., Sun X., Wang Y., Zheng X., Wang Y., Pan G. (2018). Binless Kernel Machine: Modeling Spike Train Transformation for Cognitive Neural Prostheses. Neural Comput..

[33] Ando T., Konishi S. Neural Network Nonlinear Regression Modeling and Information Criteria. Proceedings of the Advances in Statistics, Combinatorics & Related Areas-selected Papers from the Scra-fim Viii-the Wollongong Conference.

[34] Kim S.P., Sanchez J.C., Rao Y.N., Erdogmus D., Carmena J.M., Lebedev M.A., Nicolelis M.A.L., Principe J.C. (2006). A comparison of optimal MIMO linear and nonlinear models for brain–machine interfaces. J. Neural Eng..

[35] Tagliabue M., Francis N., Hao Y., Duret M., Brochier T., Riehle A., Maier M.A., Eskiizmirliler S. Estimation of two-digit grip type and grip force level by frequency decoding of motor cortex activity for a BMI application. Proceedings of the International Conference on Advanced Robotis (ICAR).

[36] Sadiq M.T., Yu X., Yuan Z., Zeming F., Rehman A.U., Ullah I., Li G., Xiao G. (2019). Motor Imagery EEG Signals Decoding by Multivariate Empirical Wavelet Transform-Based Framework for Robust Brain–Computer Interfaces. IEEE Access.

[37] Willett F.R., Avansino D.T., Hochberg L.R., Henderson J.M., Shenoy K.V. (2021). High-performance brain-to-text communication via handwriting. Nature.

[38] Wang P., Jiang A., Liu X., Shang J., Zhang L. (2018). LSTM-Based EEG Classification in Motor Imagery Tasks. IEEE Trans. Neural Syst. Rehabil. Eng..

[39] Asgharpour M., Foodeh R., Daliri M.R. (2021). Regularized Kalman filter for brain-computer interfaces using local field potential signals. J. Neurosci. Methods.

[40] Irwin Z.T., Schroeder K.E., Vu P.P., Bullard A.J., Tat D.M., Nu C.S., Vaskov A., Nason S.R., Thompson D.E., Bentley J.N. (2017). Neural Control of finger movement via intracortical brain-machine interface. J. Neural Eng..

[41] Simon D. (2006). Optimal State Estimation: Kalman, H∞ and Nonlinear Approaches.

[42] Schick I., Mitter S.K. (1994). Robust recursive estimation in the presence of heavy-tailed observation noise. Ann. Stat..

[43] Principe J.C. (2010). Information Theoretic Learning: Renyi’s Entropy and Kernel Perspectives.

[44] Liu W., Pokharel P.P., Principe J.C. (2007). Correntropy: Properties and applications in non-Gaussian signal processing. IEEE Trans. Signal Process..

[45] Santamaria I., Pokharel P.P., Principe J.C. (2006). Generalized correlation function: Definition, properties, and application to blind equalization. IEEE Trans. Signal Process..

[46] Singh A., Principe J.C. Using correntropy as a cost function in linear adaptive filters. Proceedings of the International Joint Conference on Neural Networks (IJCNN).

[47] Shi L., Lin Y. (2014). Convex combination of adaptive filters under the maximum correntropy criterion in impulsive interference. IEEE Signal Process. Lett..

[48] Liu X., Qu H., Zhao J., Chen B. (2017). State space maximum correntropy filter. Signal Process..

[49] He R., Hu B.G., Zheng W.S., Kong X.W. (2011). Robust principal component analysis based on maximum correntropy criterion. IEEE Trans. Image Process..

[50] He R., Zheng W.S., Hu B.G. (2011). Maximum correntropy criterion for robust face recognition. IEEE Trans. Pattern Anal. Mach. Intell..

[51] Du B., Tang X., Wang Z., Zhang L., Tao D. (2019). Robust Graph-Based Semisupervised Learning for Noisy Labeled Data via Maximum Correntropy Criterion. IEEE Trans. Cybern..

[52] Mandanas F.D., Kotropoulos C.L. (2017). Robust Multidimensional Scaling Using a Maximum Correntropy Criterion. IEEE Trans. Signal Process..

[53] Bretscher O. (2009). Linear Algebra with Applications: Fourth Edition.

[54] Singh A., Principe J.C. A closed form recursive solution for maximum correntropy training. Proceedings of the IEEE International Conference on Acoustics, Speech and Signal Processing (ICASSP).

[55] Chen B., Liu X., Zhao H., Principe J.C. (2017). Maximum correntropy Kalman filter. Automatica.

[56] Hampel F.R., Ronchetti E.M., Rousseeuw P.J., Stahel W.A. (1986). Robust Statistics: The Approach Based on Influence Functions.

[57] Huber P.J., Ronchetti E.M. (2009). Robust Statistics.

[58] Protter M.H., Morrey C.B. (1977). A First Course in Real Analysis.

[59] Taylor A.E. (1952). L’Hospital’s Rule. Am. Math. Mon..

[60] Agarwal R.P., Meehan M., Regan D.O. (2001). Fixed Point Theory and Applications.

[61] Chen B., Wang J., Zhao H., Zheng N., Principe J.C. (2015). Convergence of a fixed-point algorithm under maximum correntropy criterion. IEEE Signal Process. Lett..

[62] Ortega J.R., Rheinboldt W.C. (1970). Iterative Solution of Nonlinear Equations in Several Variables.

[63] Liu X., Shen X., Chen S., Zhang X., Huang Y., Wang Y., Wang Y. (2021). Hierarchical Dynamical Model for Multiple Cortical Neural Decoding. Neural Comput..

[64] Carmena J.M., Lebedev M.A., Crist R.E., O’Doherty J.E., Santucci D.M., Dimitrov D.F., Patil P.G., Henriquez C.S., Nicolelis M.A.L. (2003). Learning to Control a Brain–Machine Interface for Reaching and Grasping by Primates. PLoS Biol..

[65] She X., Liao Y., Li H., Zhang Q., Wang Y., Zheng X. Clustering and observation on neuron tuning property for brain machine interfaces. Proceedings of the International Conference on Multisensor Fusion and Information Integration for Intelligent Systems (MFI).

[66] Zhang Z., Chen S., Yang Z., Wang Y. Tracking the Time Varying Neural Tuning via Adam on Point Process Observations. Proceedings of the 40th Annual International Conference of the IEEE Engineering in Medicine and Biology Society (EMBC).

[67] Kingma D.P., Ba J.L. Adam: A Method for Stochastic Optimization. Proceedings of the International Conference on Learning Representations (ICLR).

[68] DiGiovanna J., Mahmoudi B., Fortes J., Principe J.C., Sanchez J.C. (2009). Coadaptive Brain-Machine Interface via Reinforcement Learning. IEEE Trans. Biomed. Eng..

[69] Arduin P., Fregnac Y., Shulz D.E., Ego-Stengel V. (2013). “Master” Neurons Induced by Operant Conditioning in Motor Cortex during a Brain-Machine Interface Task. J. Neurosci..

[70] Boi F., Moraitis T., Feo V.D., Diotalevi F., Bartolozzi C., Indiveri G., Vato A. (2016). A Bidirectional Brain-Machine Interface Featuring a Neuromorphic Hardware Decoder. Fronriers Neurosci..

[71] Simeral J.D., Kim S.P., Black M.J., Donoghue J.P., Hochberg L.R. (2011). Neural control of cursor trajectory and click by a human with tetraplegia 1000 days after implant of an intracortical microelectrode array. J. Neural Eng..

[72] Rao C. (1973). Linear Statistical Inference and its Applications.

